# Comprehensive Insight into Lichen Planus Immunopathogenesis

**DOI:** 10.3390/ijms24033038

**Published:** 2023-02-03

**Authors:** Marijana Vičić, Nika Hlača, Marija Kaštelan, Ines Brajac, Vlatka Sotošek, Larisa Prpić Massari

**Affiliations:** 1 Department of Dermatovenereology, Medical Faculty, University of Rijeka, Clinical Hospital Center Rijeka, Krešimirova 42, 51000 Rijeka, Croatia; 2 Department of Anesthesiology, Reanimation and Intensive Care, Medical Faculty, University of Rijeka, Clinical Hospital Center Rijeka, Tome Strižića 3, 51000 Rijeka, Croatia

**Keywords:** antibodies, dendritic cells, etiology, immunopathogenesis, keratinocytes, lichen planus, macrophages, mast cells, NK-cells, T lymphocytes

## Abstract

Lichen planus is a chronic disease affecting the skin, appendages, and mucous membranes. A cutaneous lichen planus is a rare disease occurring in less than 1% of the general population, while oral illness is up to five times more prevalent; still, both forms equally impair the patient’s quality of life. The etiology of lichen planus is not entirely understood. Yet, immune-mediated mechanisms have been recognized since environmental factors such as hepatitis virus infection, mechanical trauma, psychological stress, or microbiome changes can trigger the disease in genetically susceptible individuals. According to current understanding, lichen planus immunopathogenesis is caused by cell-mediated cytotoxicity, particularly cytotoxic T lymphocytes, whose activity is further influenced by Th1 and IL-23/Th-17 axis. However, other immunocytes and inflammatory pathways complement these mechanisms. This paper presents a comprehensive insight into the actual knowledge about lichen planus, with the causal genetic and environmental factors being discussed, the immunopathogenesis described, and the principal effectors of its inflammatory circuits identified.

## 1. Introduction

Lichen planus (LP) is a chronic, immune-mediated, mucocutaneous inflammatory disorder [1]. The classic disease is defined by the so-called “rule six P”, which summarizes the skin lesions’ characteristics, i.e., planar, purple, polygonal, pruritic, papule and plaque [2]. Cutaneous LP is a rare dermatosis whose prevalence ranges from 0.22 to 1% and involves equally people of both sexes and different races. Instead, the more frequent oral disease occurs in 2 to 5% of the general population and is twice as common in women [3,4,5,6]. Although LP primarily includes the skin and oral mucosa, other mucous membranes and skin appendages, such as nails and hair, can also be damaged [5]. LP usually appears in middle-aged adults from 30 to 60 years of age and rarely affects other age groups [4].

Although the cutaneous disease has numerous clinical variants, its typical form is the classic LP, presenting with small, sharply demarcated, flattened and polygonally shaped erythematous-livid papules, which may coalesce as the disease progresses [4]. A prominent feature of LP is epidermal hypergranulosis, manifested as whitish reticulate structures or Wickham’s striae on the lesions’ surface [2]. A classic LP usually presents as localized form, with skin changes confined to the extremities, especially the wrists, ankles, dorsal surfaces of the hands and feet, and the lumbar region [2], or less commonly as a generalized condition, involving the entire body, including the oral and anogenital mucosa [4]. Despite the impressive clinical presentation, the latter has a good prognosis, marked by a spontaneous withdrawal within two years of onset [2]. Skin LP is generally followed by severe pruritus, whose intensity corresponds to the affected surface but lacks visible scratches or secondary infections [5].

Oral LP (OLP) occurs in about 70% of patients with classic skin disease, while in 20–30% of patients it represents the only manifestation of the illness. Although it can appear in six different forms, the reticular one predominates and is followed by the erosive form, characterized by pain, chronic, recalcitrant course, and possible malignant transformation into squamous cell carcinoma [6,7]. In addition, LP can affect the mucous membranes of the external genitalia, vagina, perianal area, urethra, conjunctiva, nose, larynx and oesophagus [5]. Lichen planopilaris (LPP) is a relatively uncommon form of the disease in which inflammation and keratotic papules surround the scalp hair follicles, eventually leading to scarring alopecia [8]. The follicular form of LP is fivefold commoner in women [4]. In 10% of patients with cutaneous LP, the disease affects few or all nails by the longitudinal ridging, splitting, thinning, and in uttermost cases, pterygium formation [9].

Recent studies have shown that patients with LP will more likely suffer from certain comorbidities [10,11]. The association of LP with a group of autoimmune diseases, namely liver disease, morphea, systemic lupus erythematosus (SLE), dermatomyositis, and Sjörgen’s syndrome, has been observed [1,10,11,12,13,14]. Its positive correlation with alopecia areata, vitiligo, thyroid diseases, especially Hashimoto’s thyroiditis, and ulcerous colitis has also been confirmed [1,14,15,16]. The mechanisms of the chronic inflammatory process contribute to the more frequent occurrence of obesity, cardiovascular diseases, dyslipidemia, and diabetes, i.e., metabolic syndrome in diseased persons [11,17,18,19]. Mental disorders, such as anxiety, depression, psychological stress and sleep disturbances, are more common in LP patients as well [20,21,22]. The results of various quality of life (QoL) questionnaires showed that feeding and sexual intercourse difficulties and pain in patients with mucosal LP, as well as itching and residual hyperpigmentation in those with the cutaneous form of the disease, lead to complications in emotional, social, and mental functioning, with significant impairment of their QoL [10,23].

## 2. Etiology of LP

**Figure 1:**
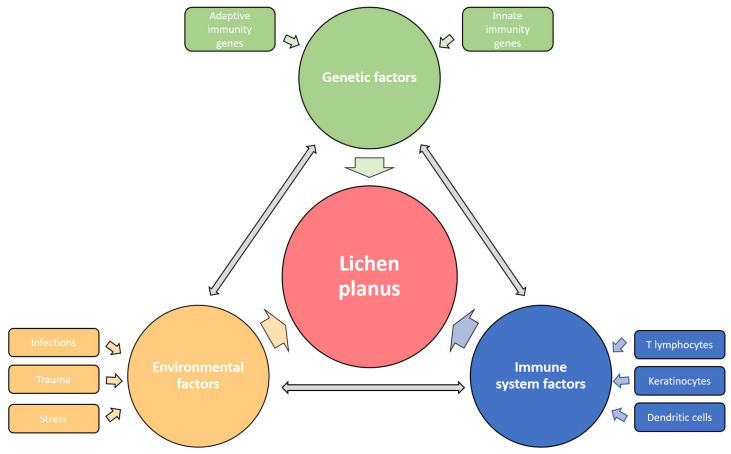
Etiological factors involved in the pathogenesis of LP. The disease affects the carrier of predisposing genes, in whom the various environmental factors trigger the immune response disorder resulting in specific LP phenotypes. The influences of genetic, environmental, and immune factors in LP development are dependent and mutually interconnected.

The cause of LP has not been fully determined; however, genetic and environmental factors are thought to play a significant role in the onset of the disease (Figure 1).

### 2.1. Genetic Factors

Genetic predisposition to disease was first suspected after noticing the LP presence in identical twins and 10% of the patients’ first relatives [2]. This observation confirmed the existence of a familial LP form, which constitutes up to 10% of all LP cases and is characterized by earlier onset, commoner relapses, treatment resistance, and oral mucosa involvement [24]. Still, genetic proneness has been observed in sporadic cases of LP. Furthermore, HLA-based susceptibility association studies identified a heterogeneous group of risk genotypes, some of which are primarily related to the familial form of the disease, such as HLA-A3, -Aw19, -B7, -B18 and -Cw8, the others such as HLA-DR1, -DRB1, -DQ1 and -Bw35 stand out in sporadic cases of cutaneous LP, while HLA-B8, -B51, -Bw57, -Bw61, are mostly expressed in OLP [2]. More detailed data on LP genetics were provided by a phenome-wide association study (PheWAS) revealing six single nucleotide polymorphisms (SNP), among which rs794275 was identified as the most significant, and HLA-DQB1 * 05:01 as the highly associated with LP [25]. Additionally, one genome-wide association study (GWAS) identified two more SNPs relevant for LP, i.e., rs884000 in the NRP2 and rs538399 in the IGFBP4 locus [26]. The conducted analyses discovered that the abovementioned polymorphisms principally affect genes with immune functions, such as Th1 response signaling molecules, i.e., TNF-α and IFN-γ, but also IL-4, IL-6, IL-10 and IL-12, IL-18, genes of oxidative stress, synthesis of thyroid hormones, prostaglandin E2, prothrombin, and NF-κB, which may impact the inflammatory mediators’ activity and cause disturbed signaling [1]. Genetic liability determines the reactivity of the patient’s skin and mucous membranes to other etiological factors.

### 2.2. Environmental Factors

Since the clinical course of the classic cutaneous LP is mainly marked by the acute beginning of one episode of the disease and its self-limiting nature, the microorganisms were often suspected as an LP causative agent. The association of LP with hepatitis C virus (HCV), which is up to thirteen times more common in LP patients, is best studied and verified by the results of several reports and meta-analyses conducted during the last decades [27,28,29,30,31]. A positive correlation between LP and HCV was observed among the populations of the Mediterranean, Germany, Japan, and the USA [32]. Although the exact role of HCV in LP development has still not been explained, viral components probably lead to host immune response dysregulation, as terminally differentiated and virus-specific CD8+ T lymphocytes have been found in the LP lesions [32,33]. Di Stasio et al. reported the improvement of OLP after direct-acting antiviral therapy, supporting the possible pathogenetic role of HCV infection in LP [33]. The increased prevalence and pathogenetic association of human papillomavirus (HPV) with OLP lesions have been proved [34], while the other possible causes include human herpesvirus 7 (HHV-7), found in infiltrating plasmacytoid dendritic cells in lesional skin biopsies [35,36,37], hepatitis B virus (HBV) [38,39], varicella-zoster virus (VZV) [40], and Epstein–Barr virus (EBV) [41]. In some cases, LP appeared or worsened following vaccination against hepatitis A and B [42,43,44], influenza [45], rabies [46], tetanus-diphtheria-pertussis [47] or SARS-CoV-2 in recent times [48,49,50]. Despite opposing views, Helicobacter pylori could contribute to LP etiopathogenesis, as it is associated with the altered function of salivary microbiome enhancing inflammation, while its eradication alleviates disease symptoms [51,52,53]. New data indicate that microbial infection could also play a role in OLP etiopathogenesis since it has been shown that a large number of bacteria and T lymphocytes were present in the affected tissue [54]. Novel fungi-based pathogenesis of OLP considers the role of *Candida* and *Malassezia* mycotypes in instigating the LP antigen expression and consequential Th17 lymphocytes’ reaction [55]. Furthermore, significant bacterial, fungal, and viral dysbiosis was detected in the saliva of these patients, with higher levels of *Solobacterium*, *Fusobacterium*, *Porphyromonas, Prevotella, Candida, Aspergillus, Alternarium*, tick-borne encephalitis virus, brochotrix bacteriophage virus and bacillus virus SPO1 and lower levels of *Streptococci, Actinobacteria* and *Firmicutes* compared to healthy controls [54,56]. Since the oral cavity is the initial part of the digestive system and forms part of the gut–skin axis, disturbances of the oral and salivary microbiome composition can directly trigger the patient’s immune system dysregulation, causing OLP [54,56].

However, in addition to microbial factors, it has been shown that OLP can be caused by contact allergens, primarily dental filling metals, such as mercury and gold [57,58,59], while its occurrence is associated with areca nut and betel quid chewing in the Far East as well [60,61]. Chemicals such as para-phenylenediamine, dimethyl fumarate and methacrylic acid esters can cause LP-like contact dermatitis [2,62]. Various drugs, especially those from the group of antihypertensives (beta-blockers and diuretics), antimalarials, antibiotics, antidiabetics, *phosphodiesterase* (PD)-1 inhibitors, and biologicals such as anti-TNF-α and dupilumab, can induce lichenoid reactions [63,64,65,66,67,68,69]. Lichen skin changes can occur due to radiation field exposure and radiotherapy [70]. Likewise, LP can be caused by mechanical injuries, such as surgical procedures or tattooing, which is confirmed by the appearance of the Köebner phenomenon in the linear form of the disease [71,72]. It has been proven that severe psychological stress can cause LP in hitherto healthy individuals, and anxiety and depression have the same effect on the onset or worsening of an existing illness [73,74,75].

According to recent research, succinate accumulates in the tissues and cells, and upregulation of the mTOR glycolysis pathway indirectly causes apoptosis, meaning metabolic change may be a significant causative factor in LP [76].

## 3. Immunopathogenesis of LP

**Figure 2:**
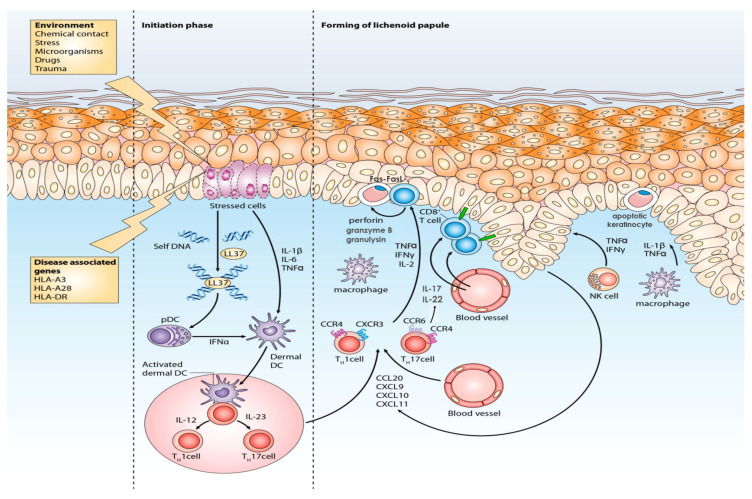
LP immunopathogenesis: major effector cells and signaling pathways included in the LP complex inflammatory network. LP inflammation begins as an antigen-directed reaction, finally resulting in the differentiation and activation of effector T lymphocytes. Th1 and Th17 lymphocytes form part of the Th1 and IL-23/Th-17 axis and influence this pathway by secreting key inflammatory cytokines such as IFN-γ and IL-17. At the same time, the key effector CD8+ lymphocytes (Tc1 and Tc17) mediate epidermal injury by the Fas-FasL and TNF-α-TNF-α receptors interaction, but primarily by engaging cytotoxic mechanisms through granule exocytosis. The release of cytotoxic molecules such as perforin, granzyme B, and granulysin causes keratinocyte apoptosis with consequent epidermal and dermal changes and the development of specific LP lesions. Other inflammatory cells such as DCs, macrophages, and NK cells also initiate and maintain the inflammatory process.

Cutaneous LP is a rare dermatosis whose etiopathogenesis has yet to be fully elucidated since animal or human investigations on this disease are generally missing. The LP pathogenesis is, in a certain part, similar to that of another related disease, psoriasis [77]. Nevertheless, the clinical diversity of these diseases indicates their immunopathogenesis particularities, as evidenced by the varied level and distribution of immune cells [78]. Reported data indicate that LP immunopathogenesis is primarily mediated by cell-mediated immune mechanisms, with T lymphocytes playing a pivotal role (Figure 2) [1,79]. The lesions’ formation following the injection of epidermotropic and autologous T lymphocytes of the LP patient into mice skin, as well as their disappearance after the administration of cellular immunity suppressants, support this thesis [80,81].

LP initiation, maintenance, and progression heavily depend on antigen-focused action, while the nonspecific and humoral mechanisms complement it to a lesser extent [1]. Based on the current understanding, LP immunopathogenesis results from the T-cell-mediated immune or autoimmune response to the exogenous or self-altered antigens presented by antigen-presenting cells (APCs) such as dendritic cells (DCs) or keratinocytes [82]. The initiation phase begins after releasing of damage-associated molecular patterns (DAMPs), such as S100A8/A9, which stimulate Toll-like receptors (TLRs). This process triggers the abundant secretion of type I interferon (IFN-α) from plasmacytoid DCs (pDCs), which is also supported by IL-1β and TNF-α produced by keratinocytes [83]. IFN-α upregulates chemokines such as CXCL9 and CXCL10 and activates inflammatory dermal DC (dDCs), which then migrate to the regional lymph nodes. There they present the antigens to naïve T lymphocytes and by releasing interleukin (IL)-12 or IL-23 promote the differentiation and expansion of T1 helper and cytotoxic (Th1 and Tc1) and T17 (Th17 and Tc17) lymphocytes’ subpopulations [83]. Interaction of keratinocytes, APCs and T lymphocytes in the early stage of the disease results in increased production of Th1 cytokines, such as IFN-γ, which is an essential event in LP [82].

In the progression phase, T1 and T17 lymphocytes, expressing skin-homing receptors, leave the bloodstream and migrate to the inflammation site, attracted by innate cells-derived cytokines and chemokines and facilitated by increased expression of adhesion molecules, such as LFA-1, ICAM-1 and VCAM-1, and basement membrane extracellular matrix proteins [82]. Memory T lymphocytes become activated upon these stimuli and respond by plentiful cytokine production, i.e., IFN-γ, TNF-α and IL-2 by Th1, or IL-17 and IL-22. Together with IL-6 and TGF-β, IL-17 stimulates additional Th17 expansion [84]. So, this complex cascade supports Th1 and Th17-related pathways, thus reinforcing the vicious circle of inflammation [84]. The ultimate effect of pro-inflammatory cytokines is the production of oxygen free radicals (ROS) that induce cell apoptosis. Considering that the prooxidant–antioxidant imbalance exists in LP, the role of oxidative stress has been postulated [85]. However, as already emphasized, Tc action is particularly significant in LP pathogenesis [76]. Namely, Tc1 and Tc17 effector cell activation promotes the caspase cascade by the interaction of TNF-α and TNF-α-R1, Fas and FasL and by unloading the cytotoxic molecules such as perforin, granzyme B, and granulysin from the cytoplasmatic granules [82]. These potent lytic particles mediate Tc-induced epidermal damage, leading to basal keratinocytes’ apoptosis and emerging characteristic LP phenotype [82]. Conducted studies indicate that granule exocytosis is a major route of cytotoxicity in humans, but others complement this mechanism at different stages of the disease [86]. Increased levels of TNF-α in sera and Fas-FasL, granzyme B, and perforin in the skin were previously found in LP patients compared to healthy controls, while recent research confirmed granulysin overexpression in the skin and peripheral blood of LP patients [87,88]. A similar finding has been demonstrated in psoriasis patients, suggesting that these chronic inflammatory dermatoses share common pathogenetic mechanisms [77,89]. While apoptosis of epithelial cells in LP is emphasized, the death of inflammatory cells is reduced, contributing to the inflammatory process’s progression [82]. Previous observations showed the importance of CD8+ and CD4+ T lymphocytes’ crosstalk since their interaction is essential for cytotoxicity activation in the affected skin [82]. After recognizing antigens expressed via MHC-I molecules by keratinocytes, CD8+ T lymphocytes express the molecule of request for cytotoxic activity (RCA) and secrete mediators to attract other inflammatory cells [90]. Meanwhile, CD4+ T lymphocytes, stimulated by DCs’ contact and IL-12, express RCA receptors and release IL-2 and IFN-γ, thus performing indirect activation and multiplication of CD8+ T lymphocytes [90].

In addition to effector cells arriving by blood, the condition is further complicated by the tissue-resident memory T cells (TRM). TRM form a local skin reservoir of chronic inflammation since they can be repeatedly activated at the previous inflammation site, causing LP reactivation and flare [91]. The regulatory T cells (Tregs) are also attracted to the skin by the abovementioned mediators. They usually play a prominent role in immunosurveillance and the suppression of effector T lymphocytes by disturbing their interaction with DCs. However, Tregs are defective in LP; therefore, they do not control the disease successfully [92]. Other immunocytes such as NK cells, macrophages, and mast cells closely interact with T lymphocytes and participate in this complex inflammatory cascade as well [83].

The action of mast cells, chemokines and matrix metalloproteinases (MMPs) contributes to nonspecific immune mechanisms [79]. In the lesional dermis, especially the basement membrane rupture area, there is an increased amount of degranulated mast cells releasing numerous mediators, such as IL-3, IL-4, IL-5, IL-6, IL-8, IL-10, IL-16, TNF-α and chymase, and RANTES [93]. RANTES (CCL5) is part of the chemokine network, which is, in addition to mast cells, produced by T lymphocytes and keratinocytes to stimulate the inflammatory cells’ migration. The importance of the CCL5-CCR5 axis has been recently pointed out by Shan et al., who reported its role in establishing LP chronicity [94]. Chemokines CXCL-9 and CXCL-10, chemokine receptors CXCR3 and CCR5, and members of the endoprotease family, MMP-2, MMP-7 and MMP-9, are also present in increased concentrations in LP [79]. Protease chymase stimulates lymphocytes’ production of MMP-9, whereby mast cells indirectly contribute to basement membrane disruption [93].

### 3.1. Main Cells Included in LP Inflammation

#### 3.1.1. Dendritic Cells

DCs play a central role in antigen presentation and T-cell response control [95]. All DC subtypes are found in increased numbers in LP lesions, indicating their contribution to the inflammatory response stimulation [82]. CD1a+ Langerhans cells (LCs) are especially numerous in the suprabasal layers of the epidermis, where they can capture and present the antigen, mediating the initial sensitivity, and then cause the secondary immune response, i.e., the lesions’ formation, and produce large amounts of IFN-α [96]. Elevated levels of CD207+ LCs have been found in LP-affected tissue [95]. Myeloid or dermal DC that can activate T lymphocytes and produce IL-12 are distributed in two types: type I (factor XIIIa+) primarily occurs in the superficial dermis, while type II (CD34+) is mainly located in the deeper dermis [81]. Plasmacytoid DCs (pDCs) are also present in the dermis, where, as the leading producers of IFN-α, they contribute to the stimulation of type 1 immune response [97].

#### 3.1.2. Macrophages

Macrophages are attracted to the lesional tissue by chemotactic signals and are found in increased numbers in LP changes [76,98,99]. Pro-inflammatory macrophages M1 clearly contribute to the pathogenesis of the disease since 60% of them are located within 125 μm of the dermo-epidermal border, i.e., in the proximate vicinity of the basement membrane [98]. CD68+ macrophages act pro-inflammatory and regulate the type of the T-cell response; therefore, they support the LP chronic inflammation by presenting antigens and producing TNF-α, IL-1β, CCL5 and MMPs [100]. Recent investigation disclosed that T cell-derived exosomes induced macrophage inflammatory protein-1α/β might drive the trafficking of CD8+ lymphocytes in OLP [101].

#### 3.1.3. Lymphocytes

The inflammatory infiltrates of the developed LP lesions composed of T lymphocytes with αβ-TCR, located at the dermal-epidermal border, causing epidermal damage is the hallmark of the disease [102]. The CD4+ T lymphocytes predominate in the dermis and CD8+ in the epidermis, especially its basal layer; simultaneously, both cell types may be part of the type 1 immune response [102]. The new immunohistochemistry study identified CD4+ and CD8+ T lymphocytes as the most abundant cells in the inflammatory infiltrate of LP skin lesions, with a ratio of 1.75:1 [99]. CD8+ T lymphocytes can recognize antigens presented as part of MHC-I molecules on keratinocytes, during routine immunosurveillance or after being attracted by keratinocytes’ chemokines [83]. Upon activation, CD8+ T lymphocytes contribute to the pathogenesis by cytotoxic mechanisms and by secretion of soluble factors that bring other inflammatory cells to the lesional area [83]. Specific LP features, which indicate the Tc importance, are intraepithelial infiltrates of these cells, especially in the area of the basement membrane and apoptotic keratinocytes as well as cytotoxic proteins found in the epidermis and connective tissue [83]. As the disease progresses, CD8+ T lymphocytes’ infiltration increases [81]. In vitro experiments have shown that isolated lesional T lymphocytes possess specific cytotoxic activity, which could be partially blocked with an anti-MHC-I monoclonal antibody [103]. These cytotoxic processes are mainly mediated by cytotoxic molecules, directed against autologous keratinocytes and can provoke irreparable damage by programmed cell death [83]. Recent studies have found CD8+ CD161+, i.e., MAIT lymphocytes, in mucosal lesions, which in response to infection release IFN-γ, TNF-α, IL-17 and IL-22 acquiring a cytotoxic phenotype, by which they kill infected cells [98]. It was noticed that a significant proportion of γδ T lymphocytes is present in lesions of LP patients vs. healthy controls [104]. However, deficient circulatory MAIT and γδ T cells expressing functional proteins and releasing cytokines may play an immunoregulatory role in the pathogenesis of OLP [105].

CD4+ T lymphocytes can recognize antigens within MHC-II molecules presented by APC or keratinocytes in the early phase of LP and, upon activation, they release cytokines that mobilize inflammatory cells but also mediate the CD8+ T lymphocytes activation and control their cytotoxicity [98]. The increased number of CD4+ T lymphocytes creates a band-like infiltrate in the subepidermal area, while they are absent in the area of the basement membrane rupture [4]. Increased CD4+ T lymphocyte infiltration is associated with a better LP response to therapy [106]. Th1 lymphocytes in the infiltrate secrete IFN-γ, IL-2, and TNF-α and activate macrophages and CD8+ T lymphocytes. Th2, Th9, and Th17 lymphocytes release IL-26, IL-22 and IL-17 and serve as drivers of the chronic tissue inflammatory response [106]. Since a significantly higher ratio of lesional Th1 and Th17 lymphocytes was found in the patients, as well as elevated serum levels of IL-17, these could play an essential role in the disease pathogenesis [106]. Previous research shows that LP patients’ peripheral blood T lymphocyte levels are significantly lower, while CD4+ and CD8+ T lymphocyte levels are reduced or unchanged [107,108]. T follicular helper (Tfh) lymphocytes have also been overexpressed in LP patients’ blood. They probably contribute to LP immunopathogenesis through abnormal modulation of B-cell proliferation and IL-21 production since Tfhs have a pivotal role in regulating humoral immunity [109].

Increased concentrations of Tregs were found in the lesional tissue and the blood of the LP patients compared to healthy controls, suggesting that a balance among the various lymphocyte subtypes could influence the clinical behavior of the disease [76,92,99]. Forkhead box protein 3 (FoxP3), predominantly expressed in the CD4+ CD25+ Treg population, plays a crucial role in Treg development. Although FoxP3+ Tregs are present in greater numbers in the lamina propria of the lesional oral mucosa and even form up to 17.7% of inflammatory infiltrate in LP skin lesions, their immunosuppressive function is impaired [92,99]. Experiments have shown that aside from Tregs dysregulation, they can be reprogrammed into pro-inflammatory cells secreting IFN-γ and IL-17 in the LP microenvironment [110]. The IL-17 expression in OLP has been positively correlated with Tregs, establishing a special relationship between Tregs and the critical producers of IL-17, i.e., Th17 lymphocytes [111]. A novel study by Xue et al. demonstrated in an animal model that the adoptive transfer of Tregs could suppress oral mucosal inflammation, offering a promising strategy in the LP treatment [112].

A possible Th17 lymphocyte plasticity change based on the surrounding milieu, as well as delicate balance and interconnections between Th1, Th17 and Treg lymphocytes, could have a decisive contribution to the LP pathogenesis [84].

#### 3.1.4. NK Cells

The CD56dimCD16- population of NK cells, which possesses cytotoxicity and TNF-α, IFN-γ, IL-22, and IL-17 production properties, was found in the lamina propria of OLP lesions and formed up to 10% of inflammatory infiltrate of LP skin lesions [99,113]. In addition, they express CXCR3, CCR5, CCR6, and ChemR23, a receptor for chemerin produced by vascular endothelial cells [113]. The function of NK cells in LP has not yet been defined. However, they probably provide an early stimulation signal for the mobilization of CD4+ and CD8+ T lymphocytes upon their arrival in the inflammation area.

#### 3.1.5. Keratinocytes

Keratinocytes are the target cells of programmed cell death or apoptosis, which is a prominent pathohistological feature of LP lesions [82]. Although in the early stages of the disease they usually begin the pathological process by exposing the antigen to CD8+ T lymphocytes within MHC-I molecules, it has been shown that they can also express MHC-II, interacting directly with CD4+ T lymphocytes [82]. They secrete type IV collagen and laminin V, which are necessary to maintain the structural integrity of the basement membrane [79]. Likewise, the survival signals sent by the intact basement membrane to keratinocytes are needed to prevent apoptosis [79]. Lesional keratinocytes, in which the process of cell death is initiated, cannot maintain the continuity of the basement membrane, so its interruption with the consequent T lymphocytes entry into the epithelium occurs [79]. Recent studies showed that apoptosis of oral keratinocytes could be caused by microRNA-122 overexpression and T cell-derived exosomes containing aberrantly expressed cytokines [114,115]. Apart from participating in apoptosis and basement membrane disruption, keratinocytes also play a role in the chronicity of the disease by secreting cytokines such as IL-1β, IL-6 and TNF-α that influence other immunocytes [83]. As skin-resident cells, keratinocytes are finally influenced by IL-17, to whose influence they readily respond [83]. New data found markedly increased filaggrin and filaggrin-2 expression in OLP lesions, highlighting the possible role of skin barrier proteins in LP pathogenesis [76].

#### 3.1.6. Mast Cells

Mast cells have been found in greater numbers in the lesional lamina propria and near the basement membrane destruction [82]. They have the ability to activate effector T lymphocytes through the expression of MHC II, CD11a, CD80 and CD86 [82]. Sixty percent of mast cells have been found degranulated in OLP lesions [79]. Upon degranulation, they release TNF-α, IL-16, proteases, CCL4, and CCL5 (RANTES) [82]. RANTES is a potent T cell chemoattractant that can act in a paracrine manner, causing repeated mast cell degranulation. This positive feedback loop amplifies and maintains LP inflammation [79]. Mast cell-derived proteases, i.e., chymase and tryptase, act as potent activators of MMPs, which participate in destroying the basement membrane [79]. Contrary to previous results, the new immunohistochemistry investigation conducted by Zychowska et al. detected an insignificant number of mast cells in the lesional inflammatory infiltrate, suggesting the minor influence of these cells in the LP pathogenesis [99].

#### 3.1.7. Neutrophils

Polymorphonuclear neutrophils (PMNs) are an essential component of innate immunity with a primary function in anti-microbial host protection. However, they can be involved in the pathogenesis of inflammatory and autoimmune diseases. The data on the PMNs’ role in LP are scarce, but a recent investigation by Khattab et al. demonstrated elevated serum levels of neutrophil activation marker calprotectin in LP patients [116]. Calprotectin is a member of the S100 protein family, expressed by PMNs, monocytes, and macrophages and is known for its chemotactic influence. According to their results, calprotectin has been proposed as the potential inflammation biomarker, which could be used in assessing the severity and progression of LP [116]. Another study conducted by Jablonska et al. showed that PMNs have the ability to form TGF-β induced neutrophil extracellular traps (NETs). Besides the potential NETs role in the LP development, they could be additionally associated with OLP malignant transformation to oral squamous cell carcinoma since, in these circumstances, they exert N2 phenotype inducing overexpression of tumor-promoting molecules such as B-cell-activating factor (BAFF) [117].

### 3.2. Main Cytokines Included in LP Inflammation

Most studies on patients’ tissue and peripheral blood samples have indicated that the Th1-immune response dominates in LP [78]. IFN-γ is a central cytokine of disease pathogenesis, which has a prominent role in LP and whose increased expression is found in subepithelial infiltrates composed of lesional CD4+ T lymphocytes and in the saliva of OLP patients [78,118]. While some authors found increased levels of IFN-γ in the patients’ serum, others did not notice differences in its expression compared to controls [119,120]. IFN-γ stimulates the expression of MHC-II molecules on keratinocytes, thereby contributing to the activation of CD8+ T lymphocytes. In addition, it promotes the expression of ICAM-1 and VCAM, affecting T lymphocyte migration and disease progression. Finally, IFN-γ activates STAT1 and, to a lesser extent, STAT3 [78]. The level of IFN-γ has been shown to correlate with the clinical manifestation of the disease since topical corticosteroid therapy reduces the number of IFN-γ+ mononuclear cells in the lesions. IFN-γ serves as the activator of the JAK-STAT signaling pathway, which affects the transcription of the inflammatory mediators’ genes and participates in the pathogenesis of numerous inflammatory diseases [121]. The off-label use of Janus kinase (JAK) inhibitors achieved excellent withdrawal of LP lesions, and further emphasized the significance of IFN-γ in LP immunopathogenesis [122,123].

The combined effect of IFN-γ with TNF-α is particularly pronounced in the cutaneous LP. TNF-α is produced by various cells, including T lymphocytes, NK cells, DC cells, macrophages, keratinocytes, and mast cells [124]. The direct association of TNF-α with the pathogenesis of the disease is evidenced by its increased levels in the lesional tissue, peripheral blood and saliva of patients with OLP which is reduced by corticosteroid, immunosuppressive and anti-TNF-α therapy [124,125,126]. TNF-α positively correlates with the NF-κB activation; stimulates the expression of LCs, adhesion molecules, and inflammatory mediators, such as RANTES and MMP-9; participates in the basal keratinocytes’ damage; and contributes to the chronic course of the disease [124]. The new comprehensive investigation of OLP patients’ saliva cytokine expression profile performed by Zhu et al. revealed significantly increased Th1-type pro-inflammatory cytokines related to NF-κB (IL-8, IL-1β, TBF-α, IL-1α, and G-CSF), and decreased IL-13 level, indicating the Th1/Th2 cytokines imbalance and emphasizing the potential NF-κB role in OLP pathogenesis [126]

IL-12, whose primary source in LP are DCs, promotes disease by stimulating the IFN-γ and IL-2 production by CD4+ T lymphocytes, ultimately leading to the activation of CD8+ T lymphocyte and NK cell cytotoxicity [82]. It is accompanied by elevated TLRs’ expression and is present in increased amounts in patients’ peripheral blood and saliva [125]. After the increased levels of IL-18 were detected in the blood of LP patients, its synergistic action with IL-12 on cytotoxicity and IFN-γ production has been confirmed [82,127].

A recent study revealed a high expression of IL-21 in LP. This cytokine stimulates the differentiation and function of CD4+/CD8+ T cells, Tfh lymphocytes and NK cells, and its expression is regulated by IL-12, IL-6 and IL-21, all of which were found upregulated in the skin of LP patients [78]. A high tissue level of myofibroblast-released IL-6 has been related to the promotion of OLP angiogenesis [128].

IL-23 is mainly produced by DCs and macrophages and is thought to be the central cytokine of many inflammatory diseases [129]. After binding to IL-23R, it contributes to STAT-3-dependent Th17 lymphocytes activation and production of IL-17, IL-22, IL-26 and CCL-20 [129]. IL-23 induces IFN-γ expression in Th17 lymphocytes, forming the highly pathogenic IFNγ+ IL-23+ T cells [129]. Overexpression of IL-23 and IL-22 was previously found in the lesional epithelium and subepithelial layer [125,130], while recently, Mardani et al. confirmed elevated IL-23 and IL-22 levels in the serum of LP patients [129,131]. IL-22 production is IL-23-dependent since IL-23, IL-18, and IL-6 increase the IL-22 production by Th22, Th17, NK cells, and innate lymphoid cells (ILCs). Thus, the upregulation of IL-22 could reflect its contribution to LP pathogenesis [131]. In addition, IL-17 overexpression has been revealed in lesional tissue and peripheral blood of LP patients [132], while high IL-17 levels in patients’ saliva showed a positive correlation with OLP clinical activity and a negative one with the amount and diversity of oral cavity microbes [133]. Some authors noticed that higher IL-17 expression in affected tissue was associated with more pronounced epidermal damage, basal membrane liquefaction, and CD4+/CD8+ T cells infiltration [118]. As presented in new data, the IL-17 and retinoic acid-related orphan receptor gamma-t (ROR-γt) expression were more pronounced in erosive than in the reticular form of OLP, concluding that this could be the reason for erosive OLP greater pathogenicity [84,134]. Although IL-17 is famous as the Th17 lymphocyte signature cytokine, it is not completely known which other immunocytes secrete it in LP. Nonetheless, one immunohistochemical analysis showed an increased expression of CD4+ and CD8+ T cells secreting IL-17 in the affected tissue and confirmed the presence of Tc lymphocytes possessing IL-17-releasing possibilities [104]. This observation indicates that Th17 and Tc17 lymphocytes are essential in the LP immunopathogenesis [104].

Excessive IL-23 and IL-17 expression in revealed LP patients compared to controls suggest a potential role of the IL-23/Th-17 axis in the LP pathogenesis. Indeed, several studies have already recognized IL-17 as a critical mediator of LP immunopathogenesis [132]. Although abundant IL-17 expression has already been observed in the diseased epithelium, the underlying mechanism of its upregulation remained unknown. However, new research revealed that NF-κB-induced renin promotes IL-17 overproduction by phosphorylation of Janus kinase 2 (JAK2) protein and subsequential activation of the signal transducer and activator of transcription 4 (STAT4), which then binds to the promoter region of the IL-17 gene in human oral keratinocytes, after translocating into the nucleus [135]. As IL-17 stimulates T lymphocyte reactions, including cytokine and chemokine synthesis, and mediates extracellular matrix damage through MMP-7 production, it likely contributes to LP pathogenesis [125]. The role of the IL-23/Th-17 axis in LP was further confirmed after off-label use of IL-23, IL-12/23, and IL-17 inhibitors led to a reduction of T1 and T17 lymphocyte cutaneous infiltrates and a significant improvement of the clinical picture [104,136]. These observations indicate that, in addition to the fundamental cytotoxic mechanisms, Th1 and IL-23/Th-17 inflammatory pathways have a central place in LP immunopathogenesis.

### 3.3. Humoral Immunity in LP Inflammation

While high serum levels of antibodies to antidesmoglein-1 and antidesmoglein-3 have been found in patients with erosive OLP and the globular IgM deposits have been confirmed by immunofluorescence studies, it is thought that B lymphocytes and humoral immunity contribute to LP to a smaller extent [97]. One immunohistochemistry analysis detected B lymphocytes and slightly fewer plasma cells in affected oral mucosa, while another confirmed a significant number of B cells in LP-affected skin [99,137]. However, the role of localized and systemic humoral immunity in OLP patients has been recently investigated by Mao et al. [107]. The direct immunofluorescence (DIF) analyses of affected mucosa showed increased IgM and IgA tissue levels, positively correlated with disease activity. Concurrently, the patients’ sera IgA antibody concentration was significantly increased; IgM, IgE, and complement C3 and C4 components significantly decreased; while IgG level did not differ compared to controls [107]. A positive correlation was also observed between IgG in serum and IgG in DIF results; CD4+ and CD4+/CD8+ ratio in serum and IgM in DIF results; and CD8+ in serum and IgM/IgA in DIF results [107]. Based on these findings, basal keratinocyte injury may result in B lymphocyte antibody production and observed changes in humoral immunity [107].

## 4. Conclusions

LP is a papular, immune-mediated dermatosis. Although it can manifest as a persistent disease affecting mucous membranes, skin appendages, and other organ systems, the classic cutaneous LP is typically a self-limiting disease with a good prognosis. The pathogenesis of LP has only recently begun to be intensively studied, and its cause is mostly undetermined. Nonetheless, according to current knowledge, cytotoxic mechanisms of cellular immunity make the critical part of LP immunopathogenesis, while the Tc influence is complemented by the action of the Th1 and Il-23/Th-17 axis. Besides the main LP effectors, i.e., Tc, Th1 and Th17 lymphocytes, other participants such as DCs, keratinocytes, NK cells, macrophages, mast cells, and Tregs also form a part of this complex inflammatory network. Recent data indicate the role of antibodies in LP pathogenesis; therefore, initiation, maintenance, and progression of LP are obviously mediated by the close interaction of both cellular and humoral immunity. Still, more investigations are needed to unravel the tangle of LP inflammation and precisely explain the nature of its immune dysregulation. Considering the therapeutic resistance of individual LP types, elucidation of immunopathogenetic mechanisms will undoubtedly contribute to developing and implementing targeted therapy that could reduce clinical symptoms and the negative impact of disease on patients’ QoL, and potentially benefit comorbidities.

## Data Availability

Not applicable.
